# Lactate-to-albumin ratio and albumin-corrected anion gap as predictors of outcome in methanol poisoning: A retrospective observational study

**DOI:** 10.1371/journal.pone.0336382

**Published:** 2025-12-04

**Authors:** İlter Ağaçkıran, Merve Ağaçkıran

**Affiliations:** 1 Department of Emergency Medicine, School of Medicine, Hitit University, Corum, Turkey; 2 Department of Emergency Medicine, Corum Erol Olcok Training and Research Hospital, Corum, Turkey; University of Rijeka Faculty of Health Studies: Sveuciliste u Rijeci Fakultet zdravstvenih studija, CROATIA

## Abstract

**Background:**

Methanol poisoning is a serious condition with high morbidity and mortality, mainly due to its metabolism to toxic formic acid. Laboratory findings, particularly albumin-corrected anion gap (ACAG) and lactate-to-albumin ratio (LAR), can lead critical in assessing the severity of toxicity. This study aims to evaluate the prognostic significance of ACAG, lactate-to-albumin ratio and other laboratory markers in methanol poisoning and to assess the impact of admission time on patient outcome.

**Methods:**

A retrospective observational study was conducted in a tertiary emergency department. Patients aged ≥18 years presenting with suspected methanol poisoning between January 2020 and December 2023 were included. Diagnosis was based on the American Academy of Clinical Toxicology (AACT) treatment criteria combined with metabolic acidosis and a suggestive history. Clinical and laboratory parameters were compared between good outcome (CPC = 1) and poor outcome (CPC ≥ 2, including deaths) groups.

**Results:**

Forty-two patients were included. The mean age was 43.45 years and 95.2% were male. The mortality rate was 35.7%. Among patients with poor outcome (n = 17), 15 died and 2 survived with severe neurological sequelae. Patients with poor outcomes had significantly lower pH and bicarbonate levels and higher AG, ACAG, lactate, and creatinine levels (p < 0.001). ACAG ≥25.6 and LAR ≥ 1.24 were the strongest prognostic markers for poor prognosis. Hypotension and osmolar gap differences were noted but were evaluated only as supportive indicators of severity.

**Conclusion:**

ACAG and LAR can lead useful prognostic markers in methanol poisoning. Patients with ACAG ≥25.6 or LAR ≥ 1.24 should be closely monitored and considered for early intensive management.

## 1. Introduction

Methanol is a highly toxic alcohol commonly used in the automotive, chemical and paint industries. Poisoning typically occurs through the consumption of alcoholic beverages that are illegally or non-commercially produced. When methanol is ingested or inhaled, it has a depressant effect on the central nervous system (CNS). However, the toxicity of methanol is mainly due to its metabolism into formaldehyde and subsequently formic acid. Formic acid is highly toxic to the optic nerve, CNS and mitochondria, and its concentration is directly related to mortality and morbidity [1].

The clinical manifestations of methanol poisoning depend on the amount ingested, the time elapsed since ingestion and the route of exposure. In the early stages, patients may present with non-specific symptoms such as nausea, vomiting, dizziness and epigastric pain, making diagnosis difficult. In the late stages (12–48 hours after exposure), methanol poisoning can result in permanent neurological deficits, blindness and death. Management of methanol toxicity depends on the extent of exposure and requires close monitoring of both laboratory parameters and clinical status. The cornerstone of treatment is the administration of antidote therapy in addition to extracorporeal treatments [2,3]. Although methanol toxicity was thought to have increased during the COVID-19 pandemic due to misinformation, the majority of cases are actually caused by the illegal sale of counterfeit alcohol [4].

High anion gap metabolic acidosis is the most prominent laboratory abnormality in methanol poisoning [5]. The serum anion gap (SAG) is a measure that reflects the balance between positively and negatively charged ions [6] and is widely used to assess metabolic acidosis. As albumin is a negatively charged molecule, variations in albumin levels can affect SAG results [7]. To address this, the concept of albumin-corrected anion gap (ACAG) has been introduced and has been shown to be a predictor of mortality in critically ill patients [8]. In toxicology, ACAG has shown promise in predicting severity in select toxicological contexts, such as lithium poisoning [9]. The lactate-to-albumin ratio is a readily quantifiable parameter that has been demonstrated to facilitate prognostication in a variety of critically ill patients, including those with gastrointestinal bleeding and sepsis in the emergency department [10–12].

This study aimed to evaluate the prognostic value of ACAG and LAR predicting mortality in methanol poisoning.

## 2. Materials and methods

### 2.1. Study Design and Setting

A single-centre retrospective observational study was conducted in the Emergency Department of a tertiary university hospital, which serves approximately 300,000 adult patients per year. Ethical approval was obtained from the Hitit University Faculty of Medicine Research Ethics Committee (approval number: 2024−119) on (date: 19.11.2024), and the study was conducted in accordance with the tenets of the Declaration of Helsinki

### 2.2. Study participants

Patients aged 18 years and over who were diagnosed with suspected methanol poisoning or methanol intoxication in the emergency department of Çorum Erol Olçok Training and Research Hospital between January 1, 2020 and December 31, 2023 were screened from the forensic registry and electronic files of patients.. For the study, study data were accessed via the hospital automation system on 25.01.2025. Methanol levels could not be measured at the study centre. The American Academy of Clinical Toxicology (AACT) ethanol treatment criteria for methanol poisoning were used for diagnosis [1]. All patients included in the study had metabolic acidosis with increased anion gap without any other cause (exclude lactic acidosis, ketoacidosis and advanced renal failure), arterial pH less than 7.3, serum bicarbonate levels less than 20 mmol/L, and a history of alcohol consumption with unknown methanol level within the last 24 hours. Osmol gap was calculated in all patients; elevated osmol gap with clinical features supported diagnosis. Other toxic alcohols (e.g., ethylene glycol) were excluded based on history, clinical presentation, and laboratory profile.

#### 2.2.1. Inclusion criteria:.

Age ≥ 18 years.

Presentation to the emergency department with suspected methanol poisoning within 24 hours of ingestion.

Presence of high anion gap metabolic acidosis (pH < 7.3, serum bicarbonate < 20 mmol/L) without another clear cause (e.g., diabetic ketoacidosis, uremic acidosis).

A history of consuming non-commercial or unknown alcoholic beverages

#### 2.2.2. Exclusion criteria.

Patients with a clear alternative cause for their metabolic acidosis (e.g., documented diabetic ketoacidosis, advanced renal failure with eGFR < 15 mL/min/1.73m²).

Patients with missing critical data for analysis (e.g., arterial blood gas, albumin levels).

Cases where the clinical history strongly suggested ingestion of another toxic alcohol (e.g., ethylene glycol) based on context or laboratory profile (e.g., presence of calcium oxalate crystals).

A total of 42 patients meeting these criteria were included in the study. A post-hoc power analysis was conducted using the G*Power 3.1 program. With 25 cases in the good outcome group and 17 cases in the poor outcome group, and an effect size (Cohen’s d) of 1.16 for the key variable ACAG, the study achieved a statistical power of 95% at an alpha error of 0.05. This indicates that the sample size, while modest, was sufficient to detect large effect sizes with high reliability.

### 2.3. Management protocol

No patient received fomepizole due to unavailability. All received ethanol infusion if indicated by AACT criteria. Hemodialysis was performed in patients with severe acidosis, visual symptoms, or renal failure. Mechanical ventilation was provided for those with respiratory failure. Rates of each intervention were recorded.

### 2.4. Data sources, measurements, and variables

The patients’ demographic characteristics and laboratory results were obtained from the hospital’s electronic and physical records. From these results, the anion gap, albumin-corrected anion gap (ACAG) and serum osmolality were calculated. The following formulae were used [13,14,15]


Anion gap (mEq/L) = sodium (mEq/L) − [chloride (mEq/L) + bicarbonate (mEq/L)]

Albumin−corrected anion gap (mEq/L) = anion gap + [2.5 × (4 – albumin, g/dL)]

Serum osmolality (mOsm/kg) = (2 × Na) + (BUN/2.8) + (glucose/18) + (ethanol/4.6)

Patient outcomes were determined using the Cerebral Perfusion Category (CPC) score [16]. Patients with a CPC score of 1 were classified as having a good outcome, whereas patients with a CPC score of 2 or higher and died were classified as having a poor outcome.

### 2.5. Statistical analysis

Data were analysed using IBM SPSS 26 software (IBM Corp., Released 2019). Normality was tested using the Shapiro-Wilk test. Depending on the distribution of the data, an independent two-sample t-test was used for normally distributed variables, whereas the Mann-Whitney U test was used for non-normally distributed variables. Categorical data were compared using Fisher’s exact test. Effect sizes were reported as Cohen’s d for continuous variables and phi coefficients for categorical variables.

The predictive performance of all blood parameters associated with poor outcome was assessed using receiver operating characteristic (ROC) analysis. Results were presented as mean ± standard deviation (SD) or median (min-max) for continuous variables and frequency (n) and percentage (%) for categorical variables. A p-value of <0.05 was considered statistically significant.

## 3. Results

The mean age of the patients included in the study was 43.45 years (age range: 18–75 years). The male:total ratio was 95.2%. The median Glasgow Coma Scale (GCS) score was found to be 10.5. A further examination of patient complaints revealed that 54.8% of patients presented with altered mental status. The mean arterial blood gas pH was 7.07, the mean anion gap was 25.28, and the mean albumin-corrected anion gap (ACAG) was 23.59. The median base deficit value was found to be 22.45. Furthermore, the median lactate level was determined to be 6.08 mmol/L, the median creatinine level was recorded as 1.2 mg/dL, and the median blood glucose level was established as 115.5 mg/dL. A significant proportion of patients, specifically 92.9%, were admitted to the intensive care unit (ICU). Furthermore, 25(59.5%) patients exhibited a favourable outcome. Of the patients, 25 were classified as having a good outcome and 17 as having a poor outcome. Of the patients with a poor outcome, 15 died, while 2 survived with severe neurological sequelae (CPC = 3). One of these patients developed permanent blindness, and the other experienced persistent cognitive impairment and motor dysfunction. Hypotension was observed in 26.2% of patients. The most common presentation time was between 12 and 18 hours after ingestion (35.7% of cases). A comprehensive overview of the demographic and clinical characteristics is provided in Table 1.

**Table 1:** Descriptive Statistics of Demographic and Clinical Characteristics.

	n(%)/Median (min.-max.)
**Age**	43.45 ± 15.28*
**Gender**	
Female	2(4.8)
Male	40(95.2)
**GCS**	10.5 (3 - 15)
**Symptom**	
Nausea-Vomiting	10(23.8)
Vission loss-Blurred vision	9(21.4)
Confusion	23(54.8)
**Blood gas pH**	7.07 ± 0.23*
**Base Deficit, mmol/L**	22.45 (2.3-36.6)
**Bicarbonate, mmol/L**	8 (2.1 - 22.5)
**PaCO2, mmHg**	30.59 ± 11.89*
**Potassium, mmol/L**	4.94 ± 1.33*
**Lactate, mmol/L**	6.08 (0.13 - 25.64)
**Creatinine, mg/dL**	1.2 (0.7 - 2.8)
**Blood glucose level, mg/dL**	115.5 (79 - 648)
**Hospitalization**	
Ward admission	3(7.1)
Intensive Care	39(92.9)
**Outcome**	
Good Outcome	25(59.5)
Poor Outcome	17(40.5)
**Anion Gap**	25.28 ± 7.64*
**Sodium, mmol/L**	135.5 (109 - 150)
**Chloride, mmol/L**	101 (74 - 110)
**Albumin, g/dL**	4.63 ± 0.63*
**Blood pressure**	
Normotension	31(73.8)
Hypotension	11(26.2)
**ACAG**	23.59 ± 7.86*
**BUN, mg/dL**	14 (7 - 42)
**Blood ethanol levels, mg/dL**	0 (0 - 33)
**Serum osmolarity**	284.7 (227.35 - 319.86)
**Hospital arrival time, hour**	
0-6	4(9.5)
6-12	12(28.6)
12-18	15(35.7)
18-24	11(26.2)

*Mean ± SD, SD: Standart devation, Median (min.-max.), GCS: Glasgow Coma Scale, ACAG: Albumin corrected anion gap, BUN: Blood urea nitrogen

Patients with poor outcomes had a significantly higher mean age (p = 0.003). Furthermore, these patients exhibited lower arterial blood gas pH and bicarbonate levels in comparison to those with favourable outcomes (p < 0.001). Conversely, anion gap and ACAG levels were found to be significantly higher in patients with poor outcomes compared to those with good outcomes (p < 0.001). Furthermore, lactate and creatinine levels were elevated, while GCS scores and base deficit values were reduced in patients with poor outcomes (p < 0.001).

A statistically significant relationship was identified between outcome status and blood pressure (p < 0.001). Among patients with hypotension, 65% exhibited a poor outcome. Although hypotension and increased osmolality were associated with mortality, these parameters were considered supportive indicators of poisoning severity rather than primary prognostic variables. The main prognostic focus of this study remained on biochemical indices, particularly the albumin-corrected anion gap (ACAG) and the lactate-to-albumin ratio (LAR), which demonstrated the strongest associations with outcome. A comprehensive analysis of all parameters associated with patient outcomes is presented in Table 2.

**Table 2:** Analysis of the relationship between outcome status and demographic and clinical characteristics.

	Good Outcome (n = 25)	Poor Outcome (n = 17)	Test statistic	p	Effect size
**Age**	37.8 ± 14.9	51.76 ± 11.92	−3.223	**0.003** ^**t**^	1.013^d^
**Gender**					
Female	2 (8)	0 (0)	–		
Male	23 (92)	17 (100)			
**GCS**	15 (3 - 15)	3 (3 - 14)	−4.305	**<0.001** ^**m**^	1.777^d^
**Symptom**					
Nausea-Vomiting	10 (40)	0 (0)	–		
Vission loss-Blurred vision	9 (36)	0 (0)			
Confusion	6 (24)	17 (100)			
**Blood gas pH**	7.22 ± 0.13	6.84 ± 0.14	9.091	**<0.001** ^**t**^	2.858^d^
**Base Deficit, mmol/L**	−12.6 (−29.7 - −2.3)	−28.8 (−36.6 - −14.5)	−4.947	**<0.001** ^**m**^	2.363^d^
**Bicarbonate, mmol/L**	13.3 (3.7 - 22.5)	5.6 (2.1 - 14.3)	−4.460	**<0.001** ^**m**^	1.897^d^
**PaCO2, mmHg**	29.74 ± 10.51	31.84 ± 13.93	−0.557	0.581^t^	0.175^d^
**Potassium, mmol/L**	4.36 ± 0.86	5.81 ± 1.45	−3.717	**0.001** ^**t**^	1.168^d^
**Lactate, mmol/L**	2.37 (0.13 - 20.77)	8.15 (4.45 - 25.64)	−3.703	**<0.001** ^**m**^	1.392^d^
**Creatinine, mg/dL**	1 (0.7 - 1.5)	1.4 (1.1 - 2.8)	−4.488	**<0.001** ^**m**^	1.920^d^
**Blood glucose level, mg/dL**	109 (79 - 330)	224 (82 - 648)	−3.063	**0.002** ^**m**^	1.073^d^
**Hospitalization**					
Ward admission	3 (12)	0 (0)	–		
Intensive Care	22 (88)	17 (100)			
**Hemodialysis**					
No	7 (28)	1 (5.9)	–	0.114^f^	0.276^φ^
1 and above	18 (72)	16 (94.1)			
**Blood pressure**					
Normotension	25 (100)	6 (35.3)	–	**<0.001** ^**f**^	0.722^φ^
Hypotension	0 (0)	11 (64.7)			
**Anion Gap**	20.6 ± 5.42	32.15 ± 4.61	−7.184	**<0.001** ^**t**^	2.258^d^
**Sodium, mmol/L**	134 (109 - 145)	137 (129 - 150)	−3.178	**0.001** ^**m**^	1.125^d^
**Chloride, mmol/L**	101 (74 - 108)	101 (92 - 110)	−0.013	0.990^m^	0.004^d^
**Albumin, mg/dL**	4.69 ± 0.53	4.53 ± 0.76	0.812	0.421^t^	0.255^d^
**ACAG**	18.8 ± 5.79	30.64 ± 4.41	−7.133	**<0.001** ^**t**^	2.242^d^
**BUN, mg/dL**	12 (7 - 32)	17 (9 - 42)	−1.208	0.227^m^	0.379^d^
**Blood ethanol levels, mg/dL**	0 (0 - 22)	0 (0 - 33)	−1.254	0.210^m^	0.394^d^
**Serum osmolarity**	279.67 (227.35 - 303.41)	294.44 (282.44 - 319.86)	−4.164	**<0.001** ^**m**^	1.677^d^
**LAR**	0.46 (0.03 - 4.72)	1.67 (0.88 - 6.25)	−3,472	**0.001** ^**m**^	1.269^d^

t: Independent samples t test, m: Mann Whitney U test, f: Fisher’s exact test, d: Cohen’s d, φ: Phi, mean ± SD, SD: Standart deviation, median (min.-max.), n (%),GCS: Glasgow Coma Scale, ACAG: Albumin corrected anion gap, BUN: Blood urea nitrogen, LAR: lactate albumin ratio

Utilising ROC analysis as the criterion, all blood parameters identified as associated with unfavourable outcomes in Table 2 were determined to possess statistically significant predictive performance (p < 0.001, p = 0.002, p = 0.001). For patients exhibiting poor outcomes, the cut-off values and their predictive performance were determined as follows: The study found that the anion gap exhibited a 92% specificity and 88.24% sensitivity, with a cut-off value of ≥ 29.4 (AUC = 0.942, 95% CI: 0.870–1.000). The albumin-corrected anion gap (ACAG) demonstrated a 84% specificity and 94.12% sensitivity, with a cut-off value of ≥ 25.6 (AUC = 0.948, 95% CI: 0.889–1.000). The lactate/albumin ratio was determined to be greater than or equal to 1.24 (AUC = 0.819, 95% CI: 0.693–0.945), with a sensitivity of 94.12% and a specificity of 64%. A comprehensive analysis of these predictive blood parameters is presented in Table 3.

**Table 3:** ROC Analysis Results for Blood Parameters Associated with Poor Outcome.

	AUC (%95 CI)	p	Cut-off	Sensitivity (%)	Specificity (%)	PPV (%)	NPV (%)	PLR	NLR
**GCS**	0.867 (0.756 - 0.979)	**<0.001**	≤ 14	100	76	73.91	100	4.17	0.00
**Blood gas pH**	0.974 (0.933–1.000)	**<0.001**	≤ 7.01	94.12	96	94.12	96	23.53	0.06
**Base Deficit**	0.954 (0.889–1.000)	**<0.001**	≤ −24.4	94.12	96	94.12	96	23.53	0.06
**Bicarbonate**	0.909 (0.821 - 0.998)	**<0.001**	≤ 8	94.12	76	72.73	95	3.92	0.08
**Potassium**	0.807 (0.656 - 0.958)	**0.001**	≥ 4.79	82.35	76	70	86.83	3.43	0.23
**Lactate**	0.840 (0.720 - 0.960)	**<0.001**	≥ 7.47	76.47	84	76.4	87	4.78	0.28
**Creatinine**	0.911 (0.827 - 0.994)	**<0.001**	≥ 1.2	94.12	76	72.73	95	3.92	0.08
**Blood glucose level**	0.781 (0.628 - 0.934)	**0.002**	≥ 222	58.82	96	90.91	77.42	14.71	0.43
**Anion Gap**	0.942 (0.870–1.000)	**<0.001**	≥ 29.4	88.24	92	88.24	92	11.03	0.13
**Sodium**	0.791 (0.648 - 0.934)	**0.002**	≥ 136	82.35	72	66.67	85.71	2.94	0.25
**ACAG**	0.948 (0.889–1.000)	**<0.001**	≥ 25.6	94.12	84	80	95.45	5.88	0.07
**Osmolarity**	0.882 (0.778 - 0.987)	**<0.001**	≥ 284.77	94.12	80	76.19	95.24	4.71	0.07
**LAR**	0.819 (0.693 - 0.945)	**0.001**	≥ 1.24	94.12	64	64	64	2.61	0.09

AUC: Area under curve, CI: Confidance interval, PPV: Positive Predictive Value, NPV: Negative Predictive Value, PLR: Positive Likelihood Ratio, NLR: Negative Likelihood Ratio, GCS: Glaskow Coma Scale, ACAG: Albumin corrected anion gap, BUN: Blood urea nitrogen, LAR: lactate albumin ratio

**Fig 1:**
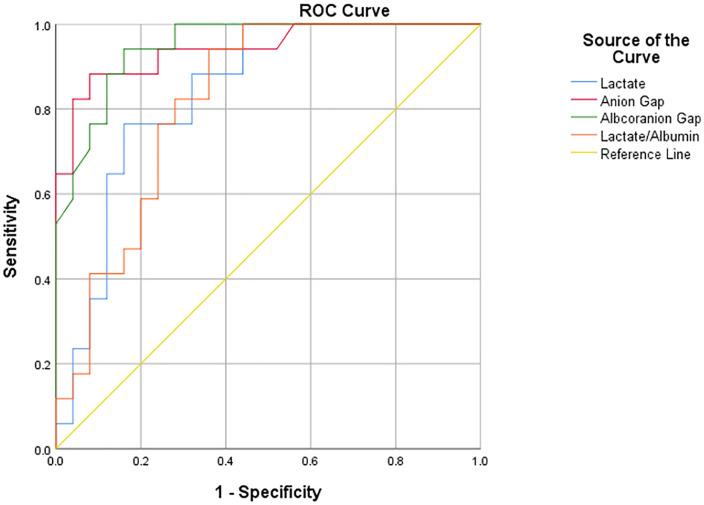
ROC curves for lactate, anion gap, ACAG (albcoranion gap), and lactate/albumin values for the outcome.

The analysis of AUC values for all blood parameters demonstrated good discriminatory ability. However, arterial blood gas pH, base deficit, bicarbonate, creatinine, anion gap, and ACAG had AUC values above 0.90, indicating that these parameters were the most powerful markers for predicting poor outcomes [17]. ROC curves for lactate, anion gap, ACAG, and LAR are illustrated in Fig 1. ack

## 4. Discussion

Methanol toxicity is a rare but potentially fatal condition if diagnosis is delayed or missed, leading to severe morbidity and mortality [1,18]. The production and consumption of home-distilled alcohol is a common practice in low-income countries, where alcohol is either prohibitively expensive or its sale and consumption are legally restricted [19]. Consequently, outbreaks of methanol toxicity are more frequently observed in countries where alcohol is illicitly sold or in low-income regions [4,20].

A number of significant parameters have been identified as predictors of mortality in methanol poisoning, including the Glasgow Coma Scale (GCS) score, severe metabolic acidosis at admission, increased anion gap, and base deficit levels. Of these, the anion gap has been identified as a particularly significant prognostic marker for methanol toxicity [18]. However, given the influence of albumin levels on the anion gap, the albumin-corrected anion gap (ACAG) was introduced to provide a more accurate reflection of unmeasured anions [14]. Hu et al. demonstrated that ACAG was superior to anion gap (AG) and albumin alone in predicting mortality among critically ill septic patients [21]. Zhong et al. also found that in critically ill patients with renal failure, ACAG levels above 20 were significantly correlated with increased mortality [8]. In the present study, ACAG levels above 25.6 in methanol intoxication cases were found to be strongly correlated with poor prognosis, and ACAG may provide comparable prognostic information to AG in this patient population.

A study by Alhusain et al. (2024) reported a mean age of 29 years and a mortality rate of 17% among methanol intoxication cases in Saudi Arabia [20]. In a retrospective study conducted in Turkey by Batur et al. (2023), the median age was 49 years, and the mortality rate was 28.57% [22]. In the present study, the mean patient age was 43.45 years, and the mortality rate was 35.7%, which is consistent with the literature.

The findings of the present study demonstrated a significant increase in mortality among patients with GCS scores of 14 or lower at admission. Concurrent studies have indicated that GCS scores of 8 or lower have been associated with increased mortality in cases of methanol poisoning [22,23]. A study conducted in Taiwan further demonstrated that each point decrease in GCS score was associated with a higher risk of mortality [24]. This phenomenon can be explained by the central nervous system depressant effects of methanol metabolites.

Although parameters such as osmolar gap and blood pressure were analyzed, they primarily reflected systemic illness severity and were not independent predictors in our model. Therefore, we limited our interpretation to laboratory indices with robust prognostic performance (ACAG and LAR). Acidosis and base deficit levels have been clearly linked to mortality in previous studies [20,22–24]. The findings of this study corroborate the observations that patients with severe acidosis and high base deficit levels exhibited a significantly higher mortality rate. In addition, a study undertaken in the Czech Republic determined that serum lactate levels exceeding 6.72 mmol/L were indicative of mortality [25]. The present study lends further support to this finding, as it was observed that lactate levels in excess of 7.47 mmol/L were indicative of a poor prognosis. Our findings regarding hyperglycemia are consistent with the study by Sanaei-Zadeh et al., who reported that elevated blood glucose levels were a strong predictor of mortality in methanol poisoning [26]. In our study, patients with poor outcomes had significantly higher glucose levels compared to those with good outcomes, suggesting that hyperglycemia may reflect the severity of metabolic stress and mitochondrial dysfunction caused by formic acid accumulation. This can be attributed to the impairment of cellular oxygenation by methanol metabolites. Furthermore, the lactate-to-albumin ratio was identified as a significant predictor of poor prognosis. This finding aligns with previous research demonstrating that the lactate-to-albumin ratio outperforms lactate alone in prognostic assessment across various conditions, including chronic kidney disease, acute pancreatitis, heart failure, and sepsis [27–29]. Since albumin is a negative acute-phase reactant, its levels may be influenced by methanol metabolites. Consequently, both ACAG and LAR are easily calculated from routine biochemical tests and can be obtained rapidly in the emergency setting. Given their prognostic benefits, they can be used to predict whether patients suspected of methanol poisoning will have a poor prognosis.

## 5. Limitations

This study has several limitations. First, it was a single-center retrospective analysis with a small sample size, which limits the generalizability of our findings. Second, methanol levels could not be measured in our center. Diagnosis was based on AACT treatment criteria, high anion gap metabolic acidosis, elevated osmol gap, compatible clinical history, and exclusion of other causes. Although this approach is commonly used in resource-limited settings, it carries a risk of misclassification bias, particularly in distinguishing methanol poisoning from other toxic alcohol ingestions.Third, exclusion of other toxic alcohols such as ethylene glycol and propylene glycol was based on clinical and basic laboratory findings rather than specific assays, which may have introduced diagnostic uncertainty. Fourth, fomepizole—recommended as the first-line antidote—was not available during the study period; all patients received ethanol infusion as antidotal therapy. This may have influenced patient outcomes.Finally, delayed presentation was common, and this factor could not be fully adjusted for in our analysis.

## 6. Conclusion

Methanol intoxication is a highly lethal condition, requiring early diagnosis and management. In our study, ACAG and LAR can lead potential prognostic marker in methanol poisoning. In centers where methanol level measurement is not available, ACAG ≥25.6 or LAR ≥ 1.24 may help identify high-risk patients who require urgent hemodialysis and intensive monitoring. These markers should be considered complementary tools rather than replacements for established clinical and laboratory assessments. Further multicenter prospective studies are needed to validate these findings.
