# Supplementary material for: Lactate-to-albumin ratio and albumin-corrected anion gap as predictors of outcome in methanol poisoning: A retrospective observational study
Source: PLoS One. 2025 Dec 4;20(12):e0336382. doi: 10.1371/journal.pone.0336382 (PMC12677794; doi:10.1371/journal.pone.0336382)
Supplement: Attachment — Submitted filename: 134039782420649360_31.docx [file pone.0336382.s001.docx]

Dear authors,

This is an interesting study that could be published with minor revisions.

1- Please specify what is meant by "poor outcome." Does it refer to deaths?

2- The mortality rate of the study was 35.7%, resulting in 15 deaths (line 214). What were the complications of the other two cases in the poor outcome group?

3- What is meant by the "sub-optimal group" (line 176)? You divided the cases into two groups, not three.

4- Sanai-Zadeh et al. showed that hyperglycemia is a strong prognostic factor for mortality in methanol poisoning. Please cite this article and compare their findings on hyperglycemia with your own.

Sanaei-Zadeh H, Esfeh SK, Zamani N, Jamshidi F, Shadnia S. Hyperglycemia is a strong prognostic factor of lethality in methanol poisoning. J Med Toxicol. 2011 Sep;7(3):189-94. doi: 10.1007/s13181-011-0142-x. PMID: 21336799; PMCID: PMC3550199.

Yours sincerely
