# Supplementary material for: Lactate-to-albumin ratio and albumin-corrected anion gap as predictors of outcome in methanol poisoning: A retrospective observational study
Source: PLoS One. 2025 Dec 4;20(12):e0336382. doi: 10.1371/journal.pone.0336382 (PMC12677794; doi:10.1371/journal.pone.0336382)
Supplement: Attachment — Submitted filename: Response Reviewers.docx [file pone.0336382.s003.docx]

**Response to Reviewers**

We sincerely thank the reviewers for their insightful comments and constructive feedback. We have carefully revised the manuscript accordingly. All changes are highlighted in yellow in the revised version.

**Reviewer 1**

The reviewer raises crucial methodological and structural points that are fundamental to the integrity of our paper. We have made extensive revisions to address these concerns.

**Comment 1:** The title does not match the research question.

**Response:** We agree with the reviewer. Our primary research question was to evaluate the **prognostic value** of ACAG and LAR for predicting mortality and poor outcomes, not just their diagnostic accuracy. The original title and presentation may have overemphasized the diagnostic test characteristics. To better reflect the study's intent, we have revised the title to:

**Revised Title:** **"Lactate-to-Albumin Ratio and Albumin-Corrected Anion Gap as Predictors of Outcome in Methanol Poisoning: A Retrospective Observational Study"**

**Comment2: The methodology states that it is a retrospective cohort study; however the results are presented as a study evaluating a diagnostic test.**

**Response: We would like to thank reviewer 1 for their valuable comments. Our study may have caused confusion, so we removed the word “cohort” and replaced it with “observational”, thinking that this would resolve the confusion. The main point we wanted to emphasise in our study is predicting mortality and poor outcomes, i.e. predicting the prognostic process.**

**Comment 3:** The inclusion and exclusion criteria are not described.

**Response:** We apologize for this oversight. We have now added a dedicated subsection under **"2.2. Study Participants"** to explicitly state the inclusion and exclusion criteria.

**Inclusion Criteria:**

1. Age ≥ 18 years.
2. Presentation to the emergency department with suspected methanol poisoning within 24 hours of ingestion.
3. Presence of high anion gap metabolic acidosis (pH < 7.3, serum bicarbonate < 20 mmol/L) without another clear cause (e.g., diabetic ketoacidosis, uremic acidosis).
4. A history of consuming non-commercial or unknown alcoholic beverages.

**Exclusion Criteria:**

1. Patients with a clear alternative cause for their metabolic acidosis (e.g., documented diabetic ketoacidosis, advanced renal failure with eGFR < 15 mL/min/1.73m²).
2. Patients with missing critical data for analysis (e.g., arterial blood gas, albumin levels).
3. Cases where the clinical history strongly suggested ingestion of another toxic alcohol (e.g., ethylene glycol) based on context or laboratory profile (e.g., presence of calcium oxalate crystals).

**Comment 4:** The sample size is not described, and if the study does not warrant it, explain why not.

**Response:** We have clarified the sample size calculation. While the study was retrospective, we performed a post-hoc power analysis to demonstrate that the sample size, though small, was sufficient to detect a large effect size with adequate power.

**“**A total of 42 patients meeting these criteria were included in the study. A post-hoc power analysis was conducted using the G*Power 3.1 program. With 25 cases in the good outcome group and 17 cases in the poor outcome group, and an effect size (Cohen's d) of 1.16 for the key variable ACAG, the study achieved a statistical power of 95% at an alpha error of 0.05. This indicates that the sample size, while modest, was sufficient to detect large effect sizes with high reliability.**”**

**Comment 5:** Many additional comments are made about other results that do not include the evaluation of a diagnostic test, such as osmolar gap or hypotension.

**Response:** We thank the reviewer for this valuable observation. We have clarified in the Results and Discussion sections that osmolar gap and hypotension were evaluated only as supportive indicators of poisoning severity, not as primary prognostic factors. The text was revised accordingly, and the discussion now focuses mainly on ACAG and LAR as the principal prognostic indices.

Revised points:

Abstract/Results: Patients with poor outcomes had significantly lower pH and bicarbonate levels and higher AG, ACAG, lactate, and creatinine levels (p <0.001). ACAG ≥25.6 and LAR ≥1.24 were the strongest prognostic markers for poor prognosis. Hypotension and osmolar gap differences were noted but were evaluated only as supportive indicators of severity.

Results: A statistically significant relationship was identified between outcome status and blood pressure (p < 0.001). Among patients with hypotension, 65% exhibited a poor outcome. Although hypotension and increased osmolality were associated with mortality, these parameters were considered supportive indicators of poisoning severity rather than primary prognostic variables. The main prognostic focus of this study remained on biochemical indices, particularly the albumin-corrected anion gap (ACAG) and the lactate-to-albumin ratio (LAR), which demonstrated the strongest associations with outcome.

Discussion: Although parameters such as osmolar gap and blood pressure were analyzed, they primarily reflected systemic illness severity and were not independent predictors in our model. Therefore, we limited our interpretation to laboratory indices with robust prognostic performance (ACAG and LAR).

**Comment 6:** The study is limited to analyzing and describing the study as a diagnostic test study, clearly specifying the gold standard and clearly justifying the usefulness of each of the diagnostic tests.

**Response:** We thank the reviewer for this helpful comment. The manuscript has been revised to clarify that our analysis aimed to assess prognostic performance, not diagnostic accuracy.

Additionally, we added an explanation in the Discussion section highlighting the clinical usefulness of ACAG and LAR as readily available biochemical markers that can assist clinicians in early predicting poor outcome. These clarifications have been incorporated into the Discussion section.

**Discussion: “Consequently, both ACAG and LAR are easily calculated from routine biochemical tests and can be obtained rapidly in the emergency setting. Given their prognostic benefits, they can be used to predict whether patients suspected of methanol poisoning will have a poor prognosis.”**

**Reviewer #2 – Comment 1:**

**Please specify what is meant by “poor outcome.” Does it refer to deaths?**

**Response:**

**Thank you for this important comment. In the revised manuscript, we have clarified that poor outcome refers to patients with Cerebral Performance Category (CPC) ≥ 2, which includes both deaths and survivors with severe neurological impairment. This definition has been added to the Methods (Section 2.4 – Data Sources, Measurements, and Variables) and briefly mentioned in the Results section.**

**“**Patient outcomes were determined using the Cerebral Perfusion Category (CPC) score ^17^. Patients with a CPC score of 1 were classified as having a good outcome, whereas patients with a CPC score of 2 or higher and died were classified as having a poor outcome.”

**Comment 2:**

The mortality rate of the study was 35.7%, resulting in 15 deaths (line 214). What were the complications of the other two cases in the poor outcome group?

**Response:**

We thank the reviewer for this question. In the poor outcome group (n = 17), 15 patients died (CPC = 5) and 2 patients survived with severe neurological sequelae. Specifically, one patient had permanent blindness and the other had persistent neurological deficits with limited cognitive recovery (both classified as CPC = 3). This clarification has been added to the Results section.

**Comment 3:**

**What is meant by the “sub-optimal group”? You divided the cases into two groups, not three.**

**Response:**

**We thank the reviewer for noticing this wording inconsistency. The term “sub-optimal group” was inadvertently used to refer to the poor outcome group (CPC ≥ 2). We have corrected this throughout the manuscript to ensure consistency. The study population was analyzed in only two categories: good outcome and poor outcome. Additionally, diagnostic accuracy, which caused confusion, has been changed to prediction performance throughout the text.**

**Comment 4:**

**Sanai-Zadeh et al. showed that hyperglycemia is a strong prognostic factor for mortality in methanol poisoning. Please cite this article and compare their findings on hyperglycemia with your own.**

**Response:**

**We appreciate this insightful suggestion. We have cited the study by Sanaei-Zadeh et al. (J Med Toxicol. 2011;7:189–94) and incorporated a comparative discussion. In our cohort, median blood glucose levels were significantly higher in the poor outcome group (224 mg/dL vs. 109 mg/dL, p = 0.002), which is consistent with Sanaei-Zadeh’s findings that hyperglycemia is associated with higher mortality in methanol poisoning. A sentence summarizing this comparison has been added to the Discussion section, and the new reference has been included in the References list.**

**Overall**

We are grateful for the reviewers’ constructive feedback, which has significantly improved the manuscript. We believe the revised version now addresses all concerns and is strengthened in clarity, methodological rigor, and clinical relevance.

Best regards.
