# Supplementary material for: Lactate-to-albumin ratio and albumin-corrected anion gap as predictors of outcome in methanol poisoning: A retrospective observational study
Source: PLoS One. 2025 Dec 4;20(12):e0336382. doi: 10.1371/journal.pone.0336382 (PMC12677794; doi:10.1371/journal.pone.0336382)
Supplement: Peer review history

# Peer review: Decision Letter 0

**DOI:** 10.1371/journal.pone.0336382.r001

**Authors:** Tanja Grubić Kezele

8 Oct 2025

Dear Dr. Ağaçkıran,

Thank you for submitting your manuscript to PLOS ONE. After careful consideration, we feel that it has merit but does not fully meet PLOS ONE’s publication criteria as it currently stands. Therefore, we invite you to submit a revised version of the manuscript that addresses the points raised during the review process.

==============================

Based on the reviewers' suggestions, the paper needs a major revision. The reviewers' comments can be found below.

==============================

Please submit your revised manuscript by Nov 22 2025 11:59PM. If you will need more time than this to complete your revisions, please reply to this message or contact the journal office at plosone@plos.org . When you're ready to submit your revision, log on to https://www.editorialmanager.com/pone/ and select the 'Submissions Needing Revision' folder to locate your manuscript file.

- A rebuttal letter that responds to each point raised by the academic editor and reviewer(s). You should upload this letter as a separate file labeled 'Response to Reviewers'.
- A marked-up copy of your manuscript that highlights changes made to the original version. You should upload this as a separate file labeled 'Revised Manuscript with Track Changes'.
- An unmarked version of your revised paper without tracked changes. You should upload this as a separate file labeled 'Manuscript'.

If you would like to make changes to your financial disclosure, please include your updated statement in your cover letter. Guidelines for resubmitting your figure files are available below the reviewer comments at the end of this letter.

If applicable, we recommend that you deposit your laboratory protocols in protocols.io to enhance the reproducibility of your results. Protocols.io assigns your protocol its own identifier (DOI) so that it can be cited independently in the future. For instructions see: https://journals.plos.org/plosone/s/submission-guidelines#loc-laboratory-protocols . Additionally, PLOS ONE offers an option for publishing peer-reviewed Lab Protocol articles, which describe protocols hosted on protocols.io. Read more information on sharing protocols at https://plos.org/protocols?utm_medium=editorial-email&utm_source=authorletters&utm_campaign=protocols .

We look forward to receiving your revised manuscript.

Kind regards,

Tanja Grubić Kezele, Ph.D., M.D.

Academic Editor

PLOS ONE

Journal Requirements:

When submitting your revision, we need you to address these additional requirements.

1. Please ensure that your manuscript meets PLOS ONE's style requirements, including those for file naming. The PLOS ONE style templates can be found at

https://journals.plos.org/plosone/s/file?id=wjVg/PLOSOne_formatting_sample_main_body.pdf and

https://journals.plos.org/plosone/s/file?id=ba62/PLOSOne_formatting_sample_title_authors_affiliations.pdf

2. We note that you have indicated that there are restrictions to data sharing for this study. PLOS only allows data to be available upon request if there are legal or ethical restrictions on sharing data publicly. For more information on unacceptable data access restrictions, please see http://journals.plos.org/plosone/s/data-availability#loc-unacceptable-data-access-restrictions.

Before we proceed with your manuscript, please address the following prompts:

a) If there are ethical or legal restrictions on sharing a de-identified data set, please explain them in detail (e.g., data contain potentially identifying or sensitive patient information, data are owned by a third-party organization, etc.) and who has imposed them (e.g., a Research Ethics Committee or Institutional Review Board, etc.). Please also provide contact information for a data access committee, ethics committee, or other institutional body to which data requests may be sent.

b) If there are no restrictions, please upload the minimal anonymized data set necessary to replicate your study findings to a stable, public repository and provide us with the relevant URLs, DOIs, or accession numbers. For a list of recommended repositories, please see

https://journals.plos.org/plosone/s/recommended-repositories. You also have the option of uploading the data as Supporting Information files, but we would recommend depositing data directly to a data repository if possible.

We will update your Data Availability statement on your behalf to reflect the information you provide.

3. Your ethics statement should only appear in the Methods section of your manuscript. If your ethics statement is written in any section besides the Methods, please delete it from any other section.

4. Please upload a copy of Figure 2, to which you refer in your text on page 14. If the figure is no longer to be included as part of the submission please remove all reference to it within the text.

If the reviewer comments include a recommendation to cite specific previously published works, please review and evaluate these publications to determine whether they are relevant and should be cited. There is no requirement to cite these works unless the editor has indicated otherwise. 

[Note: HTML markup is below. Please do not edit.]

Reviewers' comments:

Reviewer's Responses to Questions

**Comments to the Author**

1. Is the manuscript technically sound, and do the data support the conclusions?

Reviewer #1: Partly

Reviewer #2: Yes

**********

2. Has the statistical analysis been performed appropriately and rigorously?

Reviewer #1: No

Reviewer #2: Yes

**********

3. Have the authors made all data underlying the findings in their manuscript fully available?

The PLOS Data policy

Reviewer #1: Yes

Reviewer #2: Yes

**********

4. Is the manuscript presented in an intelligible fashion and written in standard English?

Reviewer #1: Yes

Reviewer #2: Yes

**********

Reviewer #1: 1. The title does not match the research question.

2. The methodology states that it is a retrospective cohort study; however the results are presented as a study evaluating a diagnostic test.

3. The inclusion and exclusion criteria are not described.

4. The sample size is not described, and if the study does not warrant it, explain why not.

5. Many additional comments are made about other results that do not include the evaluation of a diagnostic test, such as osmolar gap or hypotension.

6. The study is limited to analyzing and describing the study as a diagnostic test study, clearly specifying the gold standard and clearly justifying the usefulness of each of the diagnostic tests.

Reviewer #2: Dear authors,

This is an interesting study that could be published with minor revisions.

1- Please specify what is meant by "poor outcome." Does it refer to deaths?

2- The mortality rate of the study was 35.7%, resulting in 15 deaths (line 214). What were the complications of the other two cases in the poor outcome group?

3- What is meant by the "sub-optimal group" (line 176)? You divided the cases into two groups, not three.

4- Sanai-Zadeh et al. showed that hyperglycemia is a strong prognostic factor for mortality in methanol poisoning. Please cite this article and compare their findings on hyperglycemia with your own.

Sanaei-Zadeh H, Esfeh SK, Zamani N, Jamshidi F, Shadnia S. Hyperglycemia is a strong prognostic factor of lethality in methanol poisoning. J Med Toxicol. 2011 Sep;7(3):189-94. doi: 10.1007/s13181-011-0142-x. PMID: 21336799; PMCID: PMC3550199.

Yours sincerely

**********

what does this mean? ). If published, this will include your full peer review and any attached files.

If you choose “no”, your identity will remain anonymous but your review may still be made public.

**Do you want your identity to be public for this peer review?** For information about this choice, including consent withdrawal, please see our Privacy Policy

Reviewer #1: No

Reviewer #2: No

**********

[NOTE: If reviewer comments were submitted as an attachment file, they will be attached to this email and accessible via the submission site. Please log into your account, locate the manuscript record, and check for the action link "View Attachments". If this link does not appear, there are no attachment files.]

While revising your submission, please upload your figure files to the Preflight Analysis and Conversion Engine (PACE) digital diagnostic tool, https://pacev2.apexcovantage.com/ . PACE helps ensure that figures meet PLOS requirements. To use PACE, you must first register as a user. Registration is free. Then, login and navigate to the UPLOAD tab, where you will find detailed instructions on how to use the tool. If you encounter any issues or have any questions when using PACE, please email PLOS at figures@plos.org . Please note that Supporting Information files do not need this step.

Attachment Submitted filename: 134039782420649360_31.docx

# Author comment: Author response to Decision Letter 1

**DOI:** 10.1371/journal.pone.0336382.r002

10 Oct 2025

Response to Reviewers

We sincerely thank the reviewers for their insightful comments and constructive feedback. We have carefully revised the manuscript accordingly. All changes are highlighted in yellow in the revised version.

Reviewer 1

The reviewer raises crucial methodological and structural points that are fundamental to the integrity of our paper. We have made extensive revisions to address these concerns.

Comment 1: The title does not match the research question.

Response: We agree with the reviewer. Our primary research question was to evaluate the prognostic value of ACAG and LAR for predicting mortality and poor outcomes, not just their diagnostic accuracy. The original title and presentation may have overemphasized the diagnostic test characteristics. To better reflect the study's intent, we have revised the title to:

Revised Title: "Lactate-to-Albumin Ratio and Albumin-Corrected Anion Gap as Predictors of Outcome in Methanol Poisoning: A Retrospective Observational Study"

Comment2: The methodology states that it is a retrospective cohort study; however the results are presented as a study evaluating a diagnostic test.

Response: We would like to thank reviewer 1 for their valuable comments. Our study may have caused confusion, so we removed the word “cohort” and replaced it with “observational”, thinking that this would resolve the confusion. The main point we wanted to emphasise in our study is predicting mortality and poor outcomes, i.e. predicting the prognostic process.

Comment 3: The inclusion and exclusion criteria are not described.

Response: We apologize for this oversight. We have now added a dedicated subsection under "2.2. Study Participants" to explicitly state the inclusion and exclusion criteria.

Inclusion Criteria:

1. Age ≥ 18 years.

2. Presentation to the emergency department with suspected methanol poisoning within 24 hours of ingestion.

3. Presence of high anion gap metabolic acidosis (pH < 7.3, serum bicarbonate < 20 mmol/L) without another clear cause (e.g., diabetic ketoacidosis, uremic acidosis).

4. A history of consuming non-commercial or unknown alcoholic beverages.

Exclusion Criteria:

1. Patients with a clear alternative cause for their metabolic acidosis (e.g., documented diabetic ketoacidosis, advanced renal failure with eGFR < 15 mL/min/1.73m²).

2. Patients with missing critical data for analysis (e.g., arterial blood gas, albumin levels).

3. Cases where the clinical history strongly suggested ingestion of another toxic alcohol (e.g., ethylene glycol) based on context or laboratory profile (e.g., presence of calcium oxalate crystals).

Comment 4: The sample size is not described, and if the study does not warrant it, explain why not.

Response: We have clarified the sample size calculation. While the study was retrospective, we performed a post-hoc power analysis to demonstrate that the sample size, though small, was sufficient to detect a large effect size with adequate power.

“A total of 42 patients meeting these criteria were included in the study. A post-hoc power analysis was conducted using the G*Power 3.1 program. With 25 cases in the good outcome group and 17 cases in the poor outcome group, and an effect size (Cohen's d) of 1.16 for the key variable ACAG, the study achieved a statistical power of 95% at an alpha error of 0.05. This indicates that the sample size, while modest, was sufficient to detect large effect sizes with high reliability.”

Comment 5: Many additional comments are made about other results that do not include the evaluation of a diagnostic test, such as osmolar gap or hypotension.

Response: We thank the reviewer for this valuable observation. We have clarified in the Results and Discussion sections that osmolar gap and hypotension were evaluated only as supportive indicators of poisoning severity, not as primary prognostic factors. The text was revised accordingly, and the discussion now focuses mainly on ACAG and LAR as the principal prognostic indices.

Revised points:

Abstract/Results: Patients with poor outcomes had significantly lower pH and bicarbonate levels and higher AG, ACAG, lactate, and creatinine levels (p <0.001). ACAG ≥25.6 and LAR ≥1.24 were the strongest prognostic markers for poor prognosis. Hypotension and osmolar gap differences were noted but were evaluated only as supportive indicators of severity.

Results: A statistically significant relationship was identified between outcome status and blood pressure (p < 0.001). Among patients with hypotension, 65% exhibited a poor outcome. Although hypotension and increased osmolality were associated with mortality, these parameters were considered supportive indicators of poisoning severity rather than primary prognostic variables. The main prognostic focus of this study remained on biochemical indices, particularly the albumin-corrected anion gap (ACAG) and the lactate-to-albumin ratio (LAR), which demonstrated the strongest associations with outcome.

Discussion: Although parameters such as osmolar gap and blood pressure were analyzed, they primarily reflected systemic illness severity and were not independent predictors in our model. Therefore, we limited our interpretation to laboratory indices with robust prognostic performance (ACAG and LAR).

Comment 6: The study is limited to analyzing and describing the study as a diagnostic test study, clearly specifying the gold standard and clearly justifying the usefulness of each of the diagnostic tests.

Response: We thank the reviewer for this helpful comment. The manuscript has been revised to clarify that our analysis aimed to assess prognostic performance, not diagnostic accuracy.

Additionally, we added an explanation in the Discussion section highlighting the clinical usefulness of ACAG and LAR as readily available biochemical markers that can assist clinicians in early predicting poor outcome. These clarifications have been incorporated into the Discussion section.

Discussion: “Consequently, both ACAG and LAR are easily calculated from routine biochemical tests and can be obtained rapidly in the emergency setting. Given their prognostic benefits, they can be used to predict whether patients suspected of methanol poisoning will have a poor prognosis.”

Reviewer #2 – Comment 1:

Please specify what is meant by “poor outcome.” Does it refer to deaths?

Response:

Thank you for this important comment. In the revised manuscript, we have clarified that poor outcome refers to patients with Cerebral Performance Category (CPC) ≥ 2, which includes both deaths and survivors with severe neurological impairment. This definition has been added to the Methods (Section 2.4 – Data Sources, Measurements, and Variables) and briefly mentioned in the Results section.

“Patient outcomes were determined using the Cerebral Perfusion Category (CPC) score 17. Patients with a CPC score of 1 were classified as having a good outcome, whereas patients with a CPC score of 2 or higher and died were classified as having a poor outcome.”

Comment 2:

The mortality rate of the study was 35.7%, resulting in 15 deaths (line 214). What were the complications of the other two cases in the poor outcome group?

Response:

We thank the reviewer for this question. In the poor outcome group (n = 17), 15 patients died (CPC = 5) and 2 patients survived with severe neurological sequelae. Specifically, one patient had permanent blindness and the other had persistent neurological deficits with limited cognitive recovery (both classified as CPC = 3). This clarification has been added to the Results section.

Comment 3:

What is meant by the “sub-optimal group”? You divided the cases into two groups, not three.

Response:

We thank the reviewer for noticing this wording inconsistency. The term “sub-optimal group” was inadvertently used to refer to the poor outcome group (CPC ≥ 2). We have corrected this throughout the manuscript to ensure consistency. The study population was analyzed in only two categories: good outcome and poor outcome. Additionally, diagnostic accuracy, which caused confusion, has been changed to prediction performance throughout the text.

Comment 4:

Sanai-Zadeh et al. showed that hyperglycemia is a strong prognostic factor for mortality in methanol poisoning. Please cite this article and compare their findings on hyperglycemia with your own.

Response:

We appreciate this insightful suggestion. We have cited the study by Sanaei-Zadeh et al. (J Med Toxicol. 2011;7:189–94) and incorporated a comparative discussion. In our cohort, median blood glucose levels were significantly higher in the poor outcome group (224 mg/dL vs. 109 mg/dL, p = 0.002), which is consistent with Sanaei-Zadeh’s findings that hyperglycemia is associated with higher mortality in methanol poisoning. A sentence summarizing this comparison has been added to the Discussion section, and the new reference has been included in the References list.

Overall

We are grateful for the reviewers’ constructive feedback, which has significantly improved the manuscript. We believe the revised version now addresses all concerns and is strengthened in clarity, methodological rigor, and clinical relevance.

Best regards.

Attachment Submitted filename: Response Reviewers.docx

# Peer review: Decision Letter 1

**DOI:** 10.1371/journal.pone.0336382.r003

**Authors:** Tanja Grubić Kezele

26 Oct 2025

<p>Lactate-to-Albumin Ratio and Albumin-Corrected Anion Gap as Predictors of Outcome in Methanol Poisoning: A Retrospective Observational Study

PONE-D-25-44567R1

Dear Dr. Ağaçkıran,

We’re pleased to inform you that your manuscript has been judged scientifically suitable for publication and will be formally accepted for publication once it meets all outstanding technical requirements.

Within one week, you’ll receive an e-mail detailing the required amendments. When these have been addressed, you’ll receive a formal acceptance letter and your manuscript will be scheduled for publication.

An invoice will be generated when your article is formally accepted. Please note, if your institution has a publishing partnership with PLOS and your article meets the relevant criteria, all or part of your publication costs will be covered. Please make sure your user information is up-to-date by logging into Editorial Manager at Editorial Manager®  and clicking the ‘Update My Information' link at the top of the page. For questions related to billing, please contact billing support .

If your institution or institutions have a press office, please notify them about your upcoming paper to help maximize its impact. If they’ll be preparing press materials, please inform our press team as soon as possible -- no later than 48 hours after receiving the formal acceptance. Your manuscript will remain under strict press embargo until 2 pm Eastern Time on the date of publication. For more information, please contact onepress@plos.org.

Kind regards,

Tanja Grubić Kezele, Ph.D., M.D.

Academic Editor

PLOS ONE

Additional Editor Comments (optional):

Reviewers' comments:

Reviewer's Responses to Questions

**Comments to the Author**

Reviewer #1: All comments have been addressed

Reviewer #2: (No Response)

**********

2. Is the manuscript technically sound, and do the data support the conclusions?

Reviewer #1: Yes

Reviewer #2: Yes

**********

3. Has the statistical analysis been performed appropriately and rigorously?

Reviewer #1: Yes

Reviewer #2: Yes

**********

4. Have the authors made all data underlying the findings in their manuscript fully available?

The PLOS Data policy

Reviewer #1: Yes

Reviewer #2: Yes

**********

5. Is the manuscript presented in an intelligible fashion and written in standard English?

Reviewer #1: Yes

Reviewer #2: Yes

**********

Reviewer #1: IF ROC CURVE ANALYSIS IS TO BE USED FOR CONCLUSIONS, THE BEST INDICATOR IS THE ANION GAP TEST CORRECTED WITH ALBUMIN. THE CONCLUSION OF THE STUDY SHOULD BE ONLY THAT.

Reviewer #2: (No Response)

**********

what does this mean? ). If published, this will include your full peer review and any attached files.

If you choose “no”, your identity will remain anonymous but your review may still be made public.

**Do you want your identity to be public for this peer review?** For information about this choice, including consent withdrawal, please see our Privacy Policy

Reviewer #1: No

Reviewer #2: No

**********

# Editor report: Acceptance letter

**DOI:** 10.1371/journal.pone.0336382.r004

**Authors:** Tanja Grubić Kezele

PONE-D-25-44567R1

PLOS ONE

Dear Dr. Ağaçkıran,

I'm pleased to inform you that your manuscript has been deemed suitable for publication in PLOS ONE. Congratulations! Your manuscript is now being handed over to our production team.

At this stage, our production department will prepare your paper for publication. This includes ensuring the following:

* All references, tables, and figures are properly cited

* All relevant supporting information is included in the manuscript submission,

* There are no issues that prevent the paper from being properly typeset

You will receive further instructions from the production team, including instructions on how to review your proof when it is ready. Please keep in mind that we are working through a large volume of accepted articles, so please give us a few days to review your paper and let you know the next and final steps.

Lastly, if your institution or institutions have a press office, please let them know about your upcoming paper now to help maximize its impact. If they'll be preparing press materials, please inform our press team within the next 48 hours. Your manuscript will remain under strict press embargo until 2 pm Eastern Time on the date of publication. For more information, please contact onepress@plos.org.

You will receive an invoice from PLOS for your publication fee after your manuscript has reached the completed accept phase. If you receive an email requesting payment before acceptance or for any other service, this may be a phishing scheme. Learn how to identify phishing emails and protect your accounts at https://explore.plos.org/phishing.

If we can help with anything else, please email us at customercare@plos.org.

Thank you for submitting your work to PLOS ONE and supporting open access.

Kind regards,

PLOS ONE Editorial Office Staff

on behalf of

Prof. dr. Tanja Grubić Kezele

Academic Editor

PLOS ONE
